# Supplementary material for: Generating dynamic gene expression patterns without the need for regulatory circuits
Source: PLoS One. 2022 May 26;17(5):e0268883. doi: 10.1371/journal.pone.0268883 (PMC9135205; doi:10.1371/journal.pone.0268883)
Supplement: S1 File — (PDF) [file pone.0268883.s001.pdf]

Supplementary Information for: Generating dynamic gene expression patterns without the need for regulatory circuits

March 23, 2022

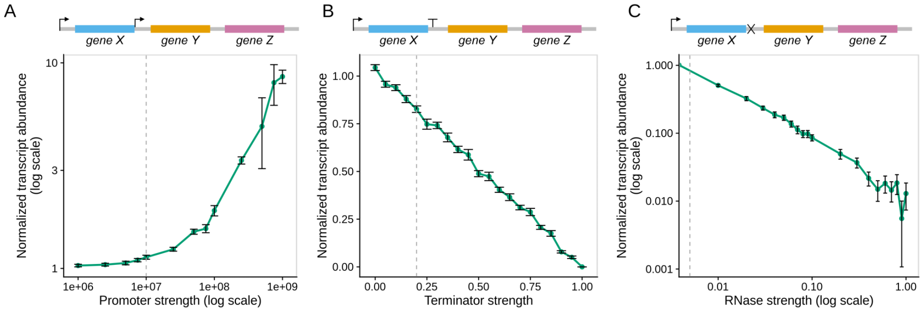

Figure S1: **Analyzing the effective range of individual regulatory element strengths.** A: The genetic architectures above the plot display the element's placement on the genome when analyzing their strengths. The line plot demonstrates a promoter's effect on gene expression at differing strengths by comparing the ratio of Gene Y over Gene X (normalized transcript abundances). The grey dashed line denotes the strength at which we decided to insert a promoter in our evolutionary simulations when an 'add' mutation is initially proposed. B: Similar to panel (A), considering transcriptional terminator strengths. C: Similar to panel (A), analyzing RNase cleavage sites. Note that a base-line level of degradation stochastically occurs from the 5' end such that in panel (B)—where the terminator strength is set to zero—the transcript abundance of gene Y ultimately may exceed gene X (resulting in a ratio that exceeds 1).

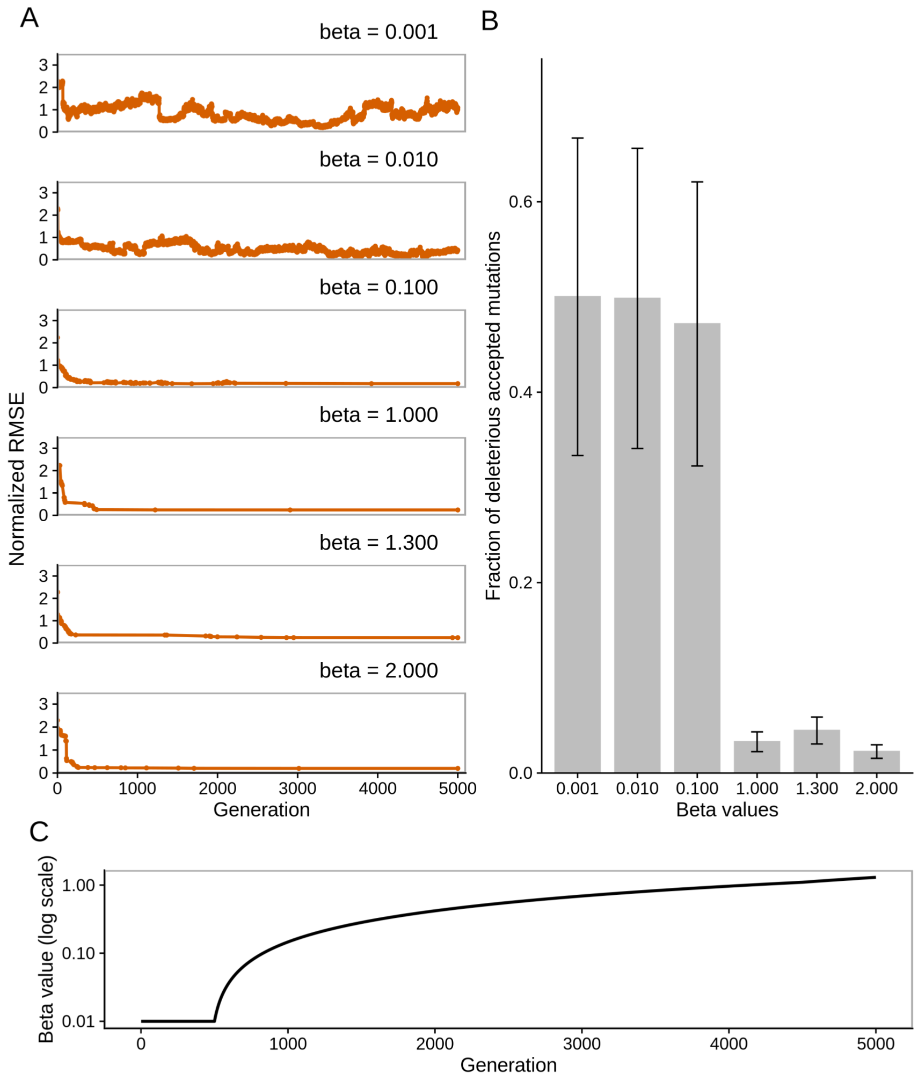

Figure S2: **Influence of β parameter on the strength of selection in our evolutionary simulations.** A: We fixed effective population size at 1000 and ran 5,000 generation simulations with the β value varying from 0.001 to 2.000. Each plots shows representatives from 10 simulations of pattern number 1. B: We summarized panel (A) by plotting the fraction of accepted mutations that were deleterious for each β value. C: We settled on an approach that varies β over the course of each simulation (simulated annealing), with this plot showing the resulting piecewise function: β remains constant for the first 10% of generations, linearly increases until 90% of the generations have passed, and then linearly increases at a different rate for the final 10% of generations.

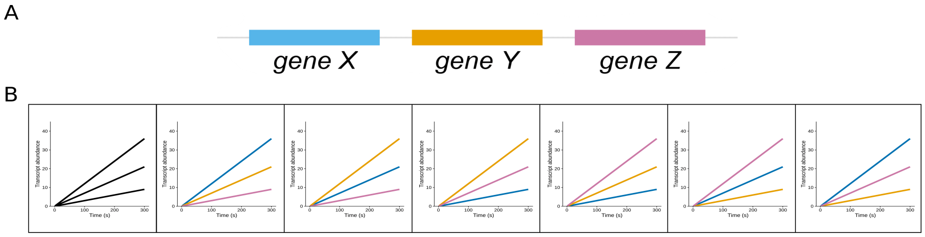

Figure S3: **Example of possible gene arrangements used in the simulations.** A: The colors in the genome are used as a legend to denote each gene: blue representing gene X, orange for gene Y, and purple for gene Z. B: The six possible gene arrangements are shown for a general pattern. The need for simulating 6 gene arrangements per general pattern arises because genomic ordering of the elements may be important and our simulation does not currently include a mutational step to swap the identity of individual elements on the genome (as might happen when recombination occurs).

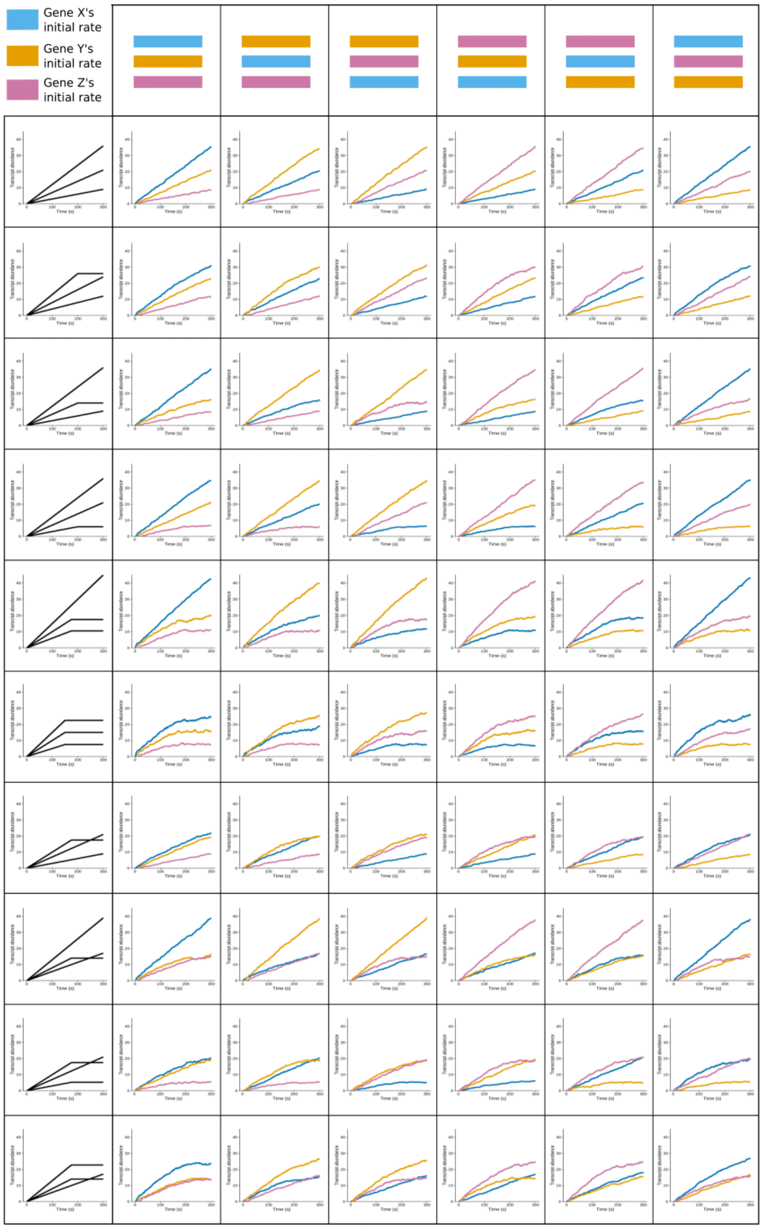

Figure S4: **Gene expression time-courses from the best genome architectures found for each target pattern.** The y-axis contains each of the general patterns shown in Fig. 3 and the x-axis shows the 6 possible gene arrangements for each pattern. Each of the depicted patterns has a normalized-RMSE value below 0.1, deeming them as successful according to our threshold.

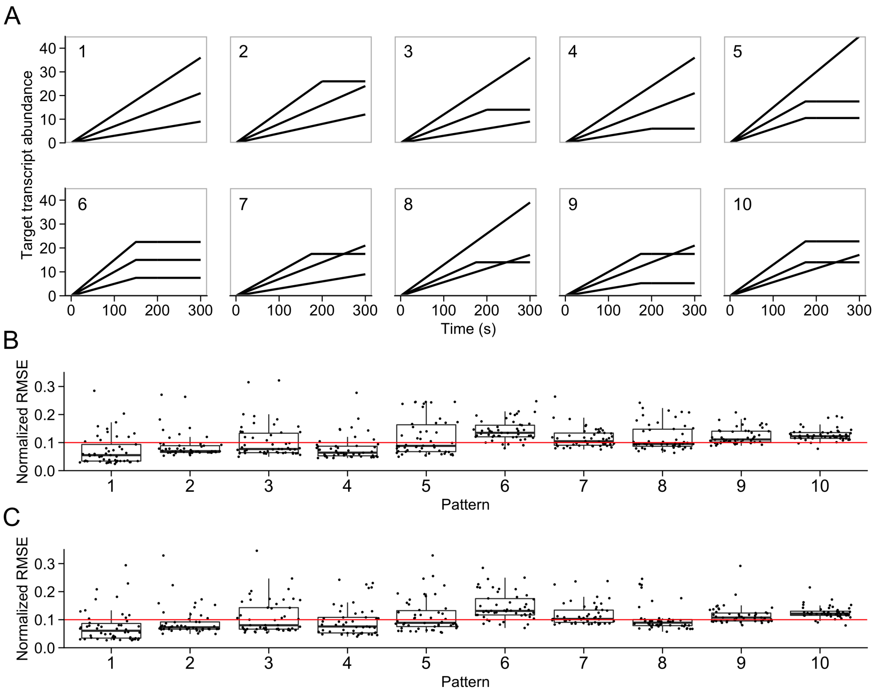

Figure S5: **Alternative fitness functions yield qualitatively similar results.** While Fig. 3 depicts results using the Fermi fitness function described in detail in the Materials and Methods, here we explored using two different functions. A: The same target patterns depicted in Fig. 3. B: Results using an exponential decline function. C: Results using a linear decline function.
