# Supplementary material for: Generating dynamic gene expression patterns without the need for regulatory circuits
Source: PLoS One. 2022 May 26;17(5):e0268883. doi: 10.1371/journal.pone.0268883 (PMC9135205; doi:10.1371/journal.pone.0268883)
Supplement: Attachment — Submitted filename: PLOS CB Revisions.pdf [file pone.0268883.s002.pdf]

Editor comments:
Thank you very much for submitting your manuscript "Generating complex patterns of gene expression without regulatory circuits" (PCOMPBIOL-D-21-00469) for consideration at PLOS Computational Biology. As with all papers peer reviewed by the journal, your manuscript was reviewed by members of the editorial board and by several independent peer reviewers. Based on the reports, we regret to inform you that we will not be pursuing this manuscript for publication at PLOS Computational Biology.

Some reviewers raised substantial concerns in terms of the model (mechanism, rationale), the evolved genome, and whether the model can be applied to explain more complex expression patterns.

The reviews are attached below this email, and we hope you will find them helpful if you decide to revise the manuscript for submission elsewhere. We are sorry that we cannot be more positive on this occasion. We very much appreciate your wish to present your work in one of PLOS's Open Access publications.

We thank the editor and reviewers for their time. We have changed the submission journal to PLOS One, and are hereby including the set of reviews and our responses to help facilitate the review process for this journal.

Reviewer comments are in black, with our responses in blue text. Most notably, we have included a new supplementary figure that includes replicated results using two different fitness functions for our Fig. 3 results. We have additionally edited the manuscript throughout (and changed the title) to downplay the complexity of the patterns that we explored so as to be careful that we are not overstating our findings. We believe that these and other alterations that we made as a result of reviewer comments have strengthened our manuscript.

Reviewer #1:
This work provides an interesting perspective on engineering of gene (or genome) circuits for multi-gene timing and levels; specifically, it builds on concepts and models from phage T7 biology, which does not rely on encoded transcriptional regulators to control phage genes. Detailed comments follow.

1) L52: Fix typo “fall off prematurely”

Fixed

2) L73: genome “injected” into host cell? How long does it take? Will the dynamics of genome entry have negligible effects on the timing of gene expression, as implied in this work?

After having gone back through our system and running test cases to assess the genome injection process, we have realized that this feature is included as a run-time option in the pinetree software but is not used by default. Consequently, our simulations and analyses do not include the genome injection step and we have removed all references to this process throughout the manuscript.

3) L71. Initial condition is four polymerase molecules? Are they host or phage RNAP? Or is the question moot? Where did they come from? What is their stability? How would the gene circuit be implemented/kickstarted in a biotech process? If the synthetic genome is delivered by phage particles, what additional constraints need to me considered (e.g., packaging signals, capsid capacity range for larger or smaller genomes)?

We specifically added:

> **“These polymerase molecules are all previously existing host-cell polymerases that do not degrade and are being co-opted by the phage. However, we note that the Pinetree platform allows for the production of phage-specific polymerases (provided that they are encoded on the phage genome) and this flexibility may allow for the production of more complicated genetic circuits---which are beyond the scope of this work.”**

We did not address specific constraints regarding the biotechnology process and actually implementing phage designs, as this is outside of the scope of the current manuscript. It is our intention to provide a basic science level proof-of-principle here, which subsequent manuscripts can build upon in a more applied / engineering manner.

4) the phage-circuit?

We were unable to find this phrase in the manuscript and are consequently unsure what the reviewer is referring to.

5) L124: why implement as stochastic simulation? Would the same or similar results be obtained for ODE implementation of the model? (For less computational time and effort?) Why not invest the computational effort and resources in exploring the genotype-fitness landscape than in running multiple stochastic implementations of the same genotype?

To address this question, we added:

> **“We chose to implement a stochastic, molecular-level simulation because ordinary differential equation-based models make a number of simplifying assumptions and thus are unable to accurately model important biological effects such as molecular collisions.”**

6) L141 Please provide the rationale for defining fitness based on the Fermi function. Why not a different function? Also tell us what is the range of the parameter beta in the fitness function; are negative values allowed?

We have added a number of changes to address this question, most notably adding an SI Fig showing results for two other fitness functions, which highlight that the choice of fitness function did not seem to be particularly important to our results. This SI fig has the following caption:

> **"Alternative fitness functions yield qualitatively similar results.** While Fig. 3 depicts results using the Fermi fitness function described in detail in the Materials and Methods, here we explored using two different functions. A: The same target patterns depicted in Fig. 3. B: Results using an exponential decline function. C: Results using a linear decline function**."**

This figure is referenced in the main text here:

> **"As a robustness check, we performed evolutionary simulations to match the same 8 patterns depicted in Fig. 3 using two different fitness functions and observed qualitatively similar results (SI Fig. 5)."**

Finally, we have added equations and relevant methodological explanations for these fitness functions to the Materials and Methods section.

7) L172 How fast is the genome "injected?" The term suggests that it is fast relative to the rates of transcription.

See our response to comment # 2, we have removed all reference to the genome injection process.

8) L190 What is the rationale for removing elements that exhibit negligible effect? Are you seeking the minimal construct to get the desired expression profile? If so, then would it be possible that two elements could play compensatory roles in the expression profile? In such a scenario, one could remove either element and the fitness would drop, but removing both and the fitness change might exhibit 'negligible effect.'

We added the following sections of text to address this concern:

> **"We did this solely so that we could investigate individual genome architecture solutions and compare the diversity of genome architectures that evolved for particular patterns."**

> **"We note that this greedy algorithm for removing individual elements neglects potential epistatic effects, such as when different regulatory elements have**

compensatory or conflicting roles. Given the small size of the genomes that we focused on here, we expect that these effects are generally minor but may nevertheless become more pronounced for larger and more complex genomes."

9) All figures are showing patterns in terms of seconds, which would be unlikely to occur for protein expression profile. Please discuss how the results (timing of protein expression) would differ if translation (protein synthesis) were implemented.

We previously included a sentence addressing this concern in our discussion:

"Finally, we focused our attention on transcript abundances but differences in translation initiation and elongation rates offer further ways to tune gene expression at the level of protein products to produce more complex dynamics."

And have edited it slightly in response to the above comment:

"Finally, we focused our attention on transcript abundances but differences in translation initiation and elongation rates offer further ways to tune gene expression at the level of protein products to produce more complex dynamics that operate over longer-time scales [7, 8, 55–58]."

10) L400 Please elaborate on what are "realistic cellular parameters"?

We previously included the line:

"We performed our simulations with realistic cellular parameters over an initial 5 minute infection period."

We have edited this so say:

"We performed our simulations with realistic cellular parameters over an initial 5 minute infection period; these parameters were previously tuned to fit empirical data on phage T7 gene expression [28] and thus represent approximately accurate values in terms of molecular rates/abundances, translocation speeds, etc."

11) L415 In the engineering of RNase sites it would be appropriate to mention earlier approaches to engineer RNA stability, for example:
Carrier, Trent A., and J. D. Keasling. "Controlling messenger RNA stability in bacteria: strategies for engineering gene expression." Biotechnology progress 13, no. 6 (1997): 699-708.

We have added this reference when discussing engineering RNA sites.

Reviewer #2:

In this manuscript, Shah et al. described an approach to design the genome of bacteriophages for generating dynamic gene expression patterns in silico. The authors simulated the stochastic gene expression of phage genomes on a platform Pinetree and implemented a simulated annealing procedure to evolve the genome to generate dynamic gene expression patterns similar to the preset pattern. The results showed that by adding, removal or alter the parameters of regulatory elements including promoters, terminators and RNase cleavage sites, a three-gene phage system can successfully generate eight different gene expression patterns, and a ten-gene system can generate a linear increase pattern with variable rates. I have several concerns about the experimental design and results of this study, which might seriously dampen the significance of this study.

(1) The authors carried out eight different gene expression patterns in Fig. 3 to investigate whether the method described in this study can generate these patterns. As the title of this manuscript described, the authors considered these patterns as “complex patterns”. However, these patterns are simply combinations of the “increase linearly” pattern and the “increase–steady” pattern of each gene. The switching time from increase to steady is same for different genes if there are more than one gene showing the “increase–steady” pattern. Thus, they can hardly be regarded as “complex patterns”. Why did the authors choose these patterns? Are they representative in phages, or are they sufficient enough for the design of synthetic phages? Are there any other patterns (such as non-monotone patterns and asynchronous patterns) that can be generated in this system? The authors should discuss these issues and also show the capacity of the system generating what kind of complex
Patterns.

We toned down some of the language throughout the manuscript, removing “complex” from the title, an appearance in the abstract, and several appearances throughout the text. We also added the following section of text:

> **“The patterns we explored were characterized by increases in absolute transcript abundances at various rates, as well as plateaus in abundance that are indicative of a steady-state balance between transcript abundance and degradation. More complex gene expression dynamics---such as gene expression delays and oscillations---will almost certainly require more complex regulatory circuitry than we allow for here. However, the range of possible dynamics that can be achieved even with combinations of plateaus and linear increases is unknown, which is why we chose to focus our efforts on these initial patterns.”**

And also added this sentence to the discussion:

> **“Finally, without allowing for more complex genetic interactions, we find it unlikely that important dynamic features such as oscillations can reliably evolve given the limited set of regulatory elements that we considered here.”**

(2) The mechanism of how these complex patterns are generated should be discussed. The phage genome structure in this study is simple. Thus, it is possible to describe the mechanism about how these patterns are generated and what kind of patterns could or could not be generated. It may be helpful to understand what happens in this system as well as guide the design of multi-gene systems in synthetic biology.

We believe that our response to this comment is covered in our response to the preceding question #1 where we discuss more about the process, rationale, and limitation behind using these particular patterns.

(3) Is it possible to manage the order of expression initiation by this model just like the delayed expression pattern shown in Fig. 5A? If so, it could expand the possible patterns to enhance the significance of this work.

We agree that delays would substantially add to the range of complexity that can be explored, and added text to highlight this very clear expression delay that only appears on Fig. 5 and discuss possible limitations of this delay mechanism:

> **“A further feature that is evident within this simulation is that the 3' most genes experience significant expression delays, presumably as a result of having evolved weaker promoters that are out-competed for polymerase molecules by the stronger 5' promoters. However, even within this 10-gene system the time-scale of these delays is modest in comparison with the 5 minute simulation time. Implementing substantial gene expression delays into realistic systems that rely solely on this delay-feature would require inserting large pieces of non-coding DNA to space apart the temporal expression of genes. While this is certainly possible, we find it likely that substantial gene expression delays can be more efficiently implemented via genetic interactions that we do not consider here.”**

(4) The authors should show more organized details about the simulation approach with tables and/or schematic diagrams, such as parameter settings and the evolution process, to help understand and reproduce the results of this study.

We have added text to re-iterate that the code is available to replicate all simulations and analyses, including all parameters.

(5) The order of Fig S1 and S2 are different from the order written in the paper.

We have corrected this error.

(6) As shown in Figure S2B, the ratio of gene Y over gene X is greater than 1 when the terminator strength is 0.00. Please give some reasonable explanations.

We added a small bit of text to the SI figure caption to explain this counterintuitive finding:

> **"Note that a base-line level of degradation stochastically occurs from the 5' end such that in panel (B)---where the terminator strength is set to zero---the transcript abundance of gene Y ultimately may exceed gene X (resulting in a ratio that exceeds 1)."**

Reviewer #3:
The manuscript of Shah et al attempts to show how a set of genes with initially identical expression profiles (ie the time course of RNA concentration over time) can be "evolved" in silico to match target expression profiles that are different from the initial condition. The evolutionary experiments are performed by "mutating" the promoters, the terminators, and RNA cleavage sites.

It is also claimed that synthetic biology circuits are unnecessary complex and that complex behaviours can be achieved via modulating the regulatory elements of individual genes.

We do not believe that we ever made such a claim, and without the reviewer pointing out where we did so, we have no way to address this comment. We took care throughout our introduction and manuscript to highlight that our goal is to explore what can be achieved via simple mechanisms for the unique cases of phages with small genomes—not to claim that anything previously done has been or is unnecessarily complex.

I am afraid that I find this work unconvincing. The target functions include expression profiles that differ from the initial conditions in their abundance, so either the expression rate goes down or degradation rate goes up; in some cases the growth curve is not linear but shows features of saturation. This is obvious to anyone in the field that such different time courses can be easily achieved by tuning promoter/terminator strength and the conclusion of the paper is therefore obvious. A slightly unobvious result is the target function that presents non-smooth time course with "kinks" in it. However, the simulation does not converge to this non-smooth function, instead showing gradual decrease in the curve.

In response to previous reviewer comments we have altered text to downplay the range of "complexity" that we explored and to ensure that we are not overstating the breadth of our results. The patterns we chose are simply proof-of-principle patterns that we find necessary to investigate before attempting to add more complex regulation or to match more complex expression patterns, which is outside the scope of the current work.

The behaviours implemented by complex synthetic circuits have included oscillations, logic gates, feedbacks, bistable systems, memory, etc. According to the claim in the introduction, all these can be achieved just by tuning gene regulatory elements. None of these complex behaviours serve as target functions in the evolutionary experiments shown in this manuscript. I

strongly doubt that indeed any of these behaviours can be achieved using the approach of the manuscript, because the approach does not enable inter-gene interactions.

Again, the reviewer states that we "claimed" something without providing a reference to where we made the claim, so we have no ability to address this comment. It is our opinion that our introduction is carefully worded and does not even vaguely make the claim that tuning regulatory elements can achieve features such as logic gates, feedbacks, bistability, *etc*. We are unsure of how the reviewer arrived at this belief and are thus unable to respond with any explicit changes.

Another major weakness of the manuscript is that the evolved genomes are not analysed, and the evolutionary solutions are not presented at all.

We note here briefly that Figs. 1, 2, 4 & 5 each display the evolutionary solutions and that Fig. 4 is dedicated solely towards analyzing the evolved genome architectures. It is possible that the reviewer is looking for an analysis of sequences, so we have clarified that the pinetree model and our evolutionary simulations do not operate on explicit "ATGC" DNA sequences, but rather on parameter files that define where in a hypothetical genome elements occur and what the numeric strength of those elements is. We have added:

> **"We emphasize here that Pinetree does not operate directly on DNA sequences but rather uses parameter file inputs that define the location and strength of individual genes, promoters, terminators, etc. on a hypothetical genome sequence."**

While performing simulations from sequence-specific information would be ideal, the vast majority of billions/trillions of possible sequence variants in promoter, terminator, etc. regions have not been characterized. The ability to accurately map from all possible sequence-level variants to regulatory element strengths is simply not possible at this stage and we have opted to specifically "mutate" numeric strengths that nevertheless have a physical location on arbitrarily defined DNA molecules.

To summarise, the current manuscript proves more or less the obvious: gene expression dynamics can be modulated via its regulatory sequences. Moreover, it fails to discuss the "solutions" found in simulations. Therefore, it does not reach the bar expected from a PLOS Computational Biology publication.

We note that our manuscript does discuss and highlight genome architecture-level solutions in detail throughout numerous figures, but the reviewer is entitled to their opinion on whether our manuscript and findings are obvious. In our view, this work has not been performed in the past, and we are unaware of studies that explicitly attempt to model phage gene expression and use evolutionary approaches to determine the range of gene expression dynamics that can be achieved using simple regulatory sequences.
